# Personalized medicine approaches for colon cancer driven by genomics and systems biology: OncoTrack

**DOI:** 10.1002/biot.201400109

**Published:** 2014-07-29

**Authors:** David Henderson, Lesley A Ogilvie, Nicholas Hoyle, Ulrich Keilholz, Bodo Lange, Hans Lehrach, on behalf of the OncoTrack Consortium

**Affiliations:** 1 Bayer Pharma AGBerlin, Germany; 2 Max Planck Institute for Molecular GeneticsBerlin, Germany; 3 Alacris TheranosticsGmbH, Berlin, Germany; 4 Roche DiagnosticsGmbH, Penzburg, Germany; 5 Charité Universitaetsmedizin Berlin, Campus Benjamin FranklinBerlin, Germany; 6 Dahlem Centre for Genome Research and Medical Systems BiologyBerlin, Germany

**Keywords:** Biomarkers, Colon cancer, Next-generation sequencing, Personalized medicine, Tumor heterogeneity

## Abstract

The post-genomic era promises to pave the way to a personalized understanding of disease processes, with technological and analytical advances helping to solve some of the world's health challenges. Despite extraordinary progress in our understanding of cancer pathogenesis, the disease remains one of the world's major medical problems. New therapies and diagnostic procedures to guide their clinical application are urgently required. OncoTrack, a consortium between industry and academia, supported by the *Innovative Medicines Initiative*, signifies a new era in personalized medicine, which synthesizes current technological advances in omics techniques, systems biology approaches, and mathematical modeling. A truly personalized molecular imprint of the tumor micro-environment and subsequent diagnostic and therapeutic insight is gained, with the ultimate goal of matching the “right” patient to the “right” drug and identifying predictive biomarkers for clinical application. This comprehensive mapping of the colon cancer molecular landscape in tandem with crucial, clinical functional annotation for systems biology analysis provides unprecedented insight and predictive power for colon cancer management. Overall, we show that major biotechnological developments in tandem with changes in clinical thinking have laid the foundations for the OncoTrack approach and the future clinical application of a truly personalized approach to colon cancer theranostics.

## 1 Introduction

Cancer is the world's leading cause of mortality, accounting for approximately 8.2 million (13% of total) deaths each year (http://globocan.iarc.fr). Across developing and developed nations alike, cancer is firmly placed on the global heath agenda, representing a major medical and socio-economic burden. Colorectal cancer (colon and rectal cancers combined) is the third most prevalent malignancy worldwide, leading to 694000 deaths in 2012 (http://globocan.iarc.fr). Although it is known that colon cancer results from a complex interplay between host-derived genetic susceptibilities and environmental factors such as diet [1], the exact etiology of the disease remains unknown, with epidemiological efforts failing to reveal concrete causal links, and large-scale dietary intervention studies failing to reduce disease risk [1].

Research into the basic mechanisms underlying the development of cancer has clearly delineated the oncogenic process, showing that the accumulation over time of multiple genetic and epigenetic changes promote tumor growth and metastasis [2, 3]; however, concomitant progress in the development of effective therapies has been much slower. This is despite a high level of investment worldwide, which has seen billions of dollars poured into finding a “cure for cancer.” In 2009 alone, cancer-related expenditure within the European Union amounted to 129 billion euros [4]. Similarly, decades of spending (109 billion dollars over 42 years) in the USA as part of the “War on Cancer” has seen cancer mortality rates decreasing by only 5% (http://www.cdc.gov/nchs/). This trend is set to continue, with projected numbers of new cases of cancer set to nearly double by 2030, reaching 22 million, accompanied by a global annual cancer death toll of 17 million (http://globocan.iarc.fr). One of the main reasons for this development is our ageing society, with a sharp rise in cancer cases in people over 60, especially in males (http://www.ons.gov.uk). Currently, over a third of cancers are identified in people over 75 years of age (http://www.ons.gov.uk).

The impending era of the global cancer “tidal wave” is set to present global health care systems with an unprecedented challenge. Currently available options for the treatment and diagnosis of cancer will, however, provide only limited protection in the face of this challenge. With drug development costs soaring and available drugs, including targeted therapies, failing to impact the mortality statistics, the race is on to find novel drugs and approaches that can respond to this global health challenge. While we have certainly won some battles in recent years, we are a long way from winning the “War on Cancer.”

At the core of these high costs and gloomy mortality statistics is the fact that even the best targeted therapies are seldom curative and generally do not lead to durable clinical responses. We consider that this is a consequence of the inherent genetic heterogeneity of tumors, their genetic instability, and the resulting ability to adapt and develop resistance under treatment. Since our diagnostic methods are often imprecise, many patients do not respond to the (often quite costly) therapies they receive, while often suffering serious side effects [5].

OncoTrack attempts to address the core problems of drug-based cancer therapies: the low response rate of patients to their therapies, as well as its inevitable consequence, the low approval rates of oncology drugs. Only about 10% of cancer drug candidates entering clinical development are granted marketing authorization. Most fail; at considerable cost, not only financially for the companies involved but also in societal terms; fewer beneficial drugs reach doctors and patients with concomitant poor health outcomes, as well as high levels of health care spending as pharmaceutical companies increase prices in an effort to recover their investment. Our program is based on two seminal developments: significant progress in our ability to analyze the molecular characteristics of individual tumors and patients (predominantly, but not exclusively, due to advancements in DNA sequencing techniques) and through the development of sophisticated computational models, which can convert this abundance of data into predictions. In a new era of personalized medicine, systems biology approaches and mathematical modeling integrate current technological advances in omics techniques to create a truly personalized model of the tumor and, potentially, of the patient.

In this review, we outline the major biotechnological advances and conceptual changes that have laid the roadmap for OncoTrack and a personalized approach to colon cancer theranostics, discussing the challenges, successes, and path ahead.

## 2 Cancer genes and genomes

Following completion of the human genome project [6, 7], which was motivated in part by the quest to understand cancer [8], sequencing has been increasingly used to characterize parts of cancer genomes, expanding from PCR-based and Sanger sequencing of key cancer genes or families (e.g. all protein kinases) [9–11] to next-generation sequencing (NGS)-based technologies. Application of these technological advances, through whole genome, whole exome, whole transcriptome, and epigenome approaches [12–14], has allowed us to obtain a more detailed overview of the mutational imprint of individual cancers and tumors.

Delineation of the full human cancer mutational landscape is revealing the complex and heterogeneous nature of human neoplasia, shifting the focus from the known key cancer genes to an expanded and flexible cancer gene mutational pool and epigenetic alterations within this landscape [15–22]. Whole exome screening has revealed key signaling pathways in breast and colon cancer [23], as well as novel epigenetic variants and other molecular phenotypes that characterize colon cancer and glioma [24, 25]. Implicit in these ground-breaking studies, however, is the realization that complete cataloguing of rare driver mutations may not be possible in many cancer types [26].

Recent efforts have taken a multi-dimensional approach, comprising exome sequencing, measurement of DNA methylation, copy number, mRNA, miRNA, non-coding RNA, and protein expression [16, 17, 27, 28], to uncover novel therapeutic possibilities for ovarian cancers [27], as well as suggesting a shared evolutionary molecular origin between cancers [16], prompting a new direction in the treatment of breast cancers. This multi-dimensional approach is also revealing a number of common mutational signatures, providing insight into the complex mechanisms underlying a range of cancer types [29, 30].

International initiatives such as the International Cancer Genome Consortium (ICGC; http://icgc.org) and The Cancer Genome Atlas (TCGA; http://cancergenome.nih.gov/) are supporting a strategic shift in the approach for understanding cancer, focusing efforts on generating a comprehensive catalog of genomic abnormalities (somatic mutations, abnormal expression of genes, and epigenetic modifications) of an estimated 25000 tumors, including those occurring in colon cancer [31]. As part of this initiative, German consortia, including the MPIMG, are sequencing 500 pediatric brain tumors [32, 33] and 250 early-onset prostate tumors. From these, we have already analyzed a small cohort, carrying out integrative genomic analyses, which revealed an androgen-driven somatic alteration landscape in early-onset prostate cancer [34].

## 3 Computer models of cancer and other biological processes

Given the multi-factorial nature of cancer and tumorigenesis, significant efforts have been focused on the development of mathematical models, seen as key to unraveling these inherent complexities [35–40], and integral to the personalization of health care [41–43].

Current models allow partial insight into the oncogenic process, providing information on the large-scale structure and development of a tumor [44], as well as the molecular processes intrinsic to the tumor cell, or, in multiscale/hybrid modeling [35, 37], attempt to combine both structural and cellular aspects.

Cancer-related models have traditionally focused on processes affecting single biological processes, such as specific pathways, facilitating enhanced understanding of tumor behavior and helping to direct therapeutic strategies. Published examples include models focusing on the epidermal growth factor receptor (EGFR), Toll-like receptor, erythropoietin (EPO), and tumor necrosis factor (TNF)-alpha mediated nuclear factor kappa B (NF-κB) signaling pathways [45–49]. Although providing unprecedented insights, key cellular influences, including the critical role of cancer-driven mutations on feedback regulation for these pathways [50], are not part of the predictive machinery of these models, limiting their impact. The ongoing challenge is therefore to develop global models that integrate all key cancer signaling and regulatory pathways to enable more focused direction of cancer therapies [51]. For further information on how these key signaling and regulatory pathways relate to the hallmarks of cancer see [2, 3].

In light of the low response rate to drugs routinely applied in cancer therapy, the application of a global model that can simulate the biological effects of drug treatment on the heterogeneous tumor and associated tissues to predict therapeutic outcome and identify biomarkers, will be an essential step toward personalization of cancer treatment.

## 4 Biomarkers: A first step forward

Over the last decade, therapeutic options for colon cancer have moved away from the application of a limited range of non-specific cytotoxic agents (5-fluoro-uracil (5-FU), irinotecan, and oxaliplatin) to include the use of selective, mechanism-based therapeutics, targeting oncoproteins that are crucial for tumor growth (reviewed in [52]). To increase the chances of a patient responding to the therapy they receive, efforts have been directed at identifying specific biomarkers, in order to detect a subgroup more likely to respond to a specific therapy (“patient stratification”).

The current clinical practice of combining selective molecular-based therapies with biomarkers, has been successful in directing several therapeutic strategies. This is exemplified by the use of human epidermal growth factor receptor 2 (HER2) status as a rationale for the selection and management of breast cancer patients suitable for treatment with trastuzumab [53]. The use of KRAS mutational status to determine response to anti-EGFR therapies (e.g. cetuximab and panitumumab) is the paradigm of stratified patient selection in colon cancer [54–57].

The original KRAS diagnostic, which assesses mutations only in codons 12 and 13 in exon 2 of the protein, improves the response rate of the corresponding combination EGRF inhibitor/irinotecan therapy in second line colon cancer from 10% in unselected patients to a still relatively meager 35% for the KRAS exon 2 wild type group [58]. In the past year, it has been reported that expanding testing to include mutations in KRAS exons 3 and 4, NRAS exons 2, 3, and 4, as well as BRAF V600E genotyping, all lead to improved response rates and are crucial for selection of patients; in particular, those who are candidates for chemotherapy in combination with anti-EGFR antibodies or in sequential treatment with the anti-angiogenic agent bevacizumab [59, 60]. These findings underscore the need for more precise molecular analyses as the basis for effective patient stratification. Because of the absence of known single driver mutations other than the *RAS*-pathway drivers, there is currently no further subdivision of colon cancer tumors into molecularly defined subgroups that would allow drug development trials with novel subgroup-tailored approaches.

Given that hundreds of genes are causally implicated in the genetic alterations contributing to cancer, focus on individual genes as diagnostics, such as KRAS and HER2, provide limited gains in therapeutic efficacy; gene expression-fingerprints are similarly limited in their applicability, highlighting the urgent requirement for a robust, systematic approach to the identification and assessment of cancer biomarkers, which would be indicative of patient response to therapy.

## 5 OncoTrack – shifting the theranostic paradigm

Over the past decades, it has become clear that tumors display a high degree of genome plasticity and molecular heterogeneity, with each tumor displaying its own dysregulated genetic, epigenetic, transcriptomic, and proteomic program [61]. Current diagnostic and therapeutic regimes are based on the organ of origin, histological grade, and the presence or absence of gene mutations [62]. There is, however, growing awareness that this approach may not provide the degree of resolution required to discriminate tumors with distinct molecular phenotypes. The stark mortality statistics are a reminder that the problem of cancer treatment requires a novel perspective, shifting the theranostic paradigm from one based on monogenic stratification to a personalized approach that focuses on a holistic understanding of the molecular landscape of individual tumor entities. Patient stratification based on a more complete description of the cancer will facilitate the selection of accurately tailored therapies, i.e. matching the “right” patient to the “right” drug. Ultimately, this will improve patient welfare, the likelihood of a positive prognosis, and reduce healthcare costs.

In the age of the “1000 dollar genome”, this goal is finally within reach. The current climate is one of technological innovation, with major leaps forward achieved not only in the accuracy and efficiency of NGS platforms and other large-scale molecular analysis techniques (proteomics and metabolomics), but also in the associated tools and concepts for analyzing the resultant omics data.

By capitalizing on these technological advances and translating them into clinical practice, we aim to redefine the paradigm of cancer theranostics through the OncoTrack project (www.oncotrack.eu), a large-scale international collaborative effort between industry, small and medium enterprises (SMEs), and academic institutions, supported by the “*Innovative Medicines Initiative*” that is focused on identifying, developing and validating biomarkers to provide a “personalized approach” to the treatment of colon cancer; a goal driven by a systems medicine approach based on in silico “Virtual Patient” models.

At the core of this challenge is the inherent complexity of the tumor/patient/drug interaction. In essence, tumors evolve as distinct entities within patients, with changes occurring at the genomic and epigenomic levels that increasingly differentiate the tumor genome from that of the patient [61, 63, 64]. Extensive genetic and phenotypic variation and plasticity exists not only between primary tumors and metastases but also within the same tumors, with an individual polyclonal tumor containing cell types that may react differently to the same treatment [61, 65, 66]. Further layers of complexity are added when considering the tumor microenvironment, immune response, and other factors such as diet, and the simple realization that every tumor arises in a patient with a unique genome. Each patient may have subtly different enzymes that activate and/or degrade the drugs he/she receives, resulting in specific reactions to the administered drugs, including different side effects [67].

The use of simple rules to predict the optimal drug for every patient, and the optimal patient collection to receive a specific drug in a clinical trial, is therefore overwhelmingly insufficient to provide the level of discrimination required from this highly intricate tumor/patient/drug system.

OncoTrack is grabbing this bull of complexity by its horns, using a strategy often implemented in situations where we face complex problems, with potentially dangerous and/or expensive consequences: we build precise computer models of the situation, based on a detailed characterization of the system. Such an approach is used in building cars, where the development time for new models of cars has dropped by two-thirds due to the extensive use of computer models (e.g. virtual crash tests), in all steps of the design and testing process. We predict the weather using detailed models based on millions of data points running on high-end computers, and train pilots on “virtual planes” (flight simulators) rather than risk letting them crash real planes with real passengers.

**Figure 1:**
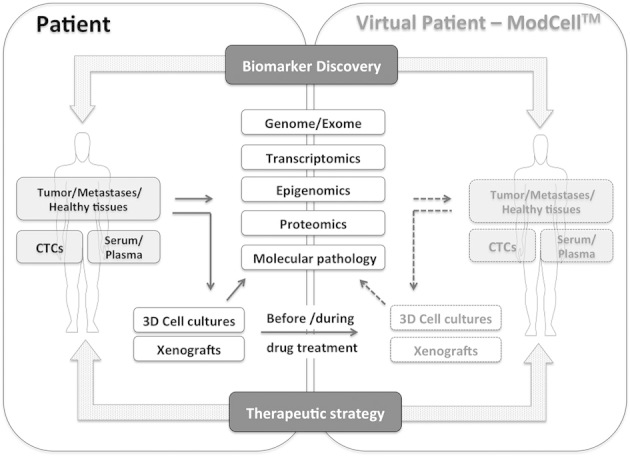
OncoTrack – shifting the theranostic paradigm. In the course of the OncoTrack program, a comprehensive and systematic molecular interrogation of the primary tumor, metastases and paired healthy tissue, comprising whole genome, exome, transcriptome, methylome, and global proteomes of colon cancer patients is carried out. In tandem, serum and plasma is collected to aid biomarker detection to support prediction of clinical outcomes. To gain insight into the response of individual tumors to drugs, 3D cell cultures of tumors and mouse xenograft models are used, supported by corresponding omics analysis (transcriptomes, proteomes, and ideally, exomes and methylomes are analyzed before and during treatments), to facilitate prediction of patient response and development of drug resistance. Resultant data from individual tumors and patients is used to seed the ModCell™ integrative systems biology predictive platform [84, 85] to focus therapeutic strategies and identify biomarkers for stratification and management of patients. In turn, computer model predictions and experimental models are used to predict tumor responders and non-responders and ultimately identify biomarkers to direct therapeutic strategy.

In a move away from traditional clinical thinking based on a stratified and correlative approach to diagnosis and therapy, OncoTrack focuses on the molecular blueprint of individual tumors (and patients) as a starting point for the computer modeling of the tumor. Tumor (and patient) are therefore defined molecularly rather than by clinical categories, in the same way drugs are primarily described by their molecular specificity. In taking such an approach, responses to any desired therapeutic scenario can be simulated, with the ultimate goal of matching every patient to the “right” drug (and, in a clinical trial, every drug to the right set of patients) either directly by the results of the modeling, or indirectly using biomarkers derived through this process (Fig. 1).

### 5.1 Attempting not to drown in data: The OncoTrack approach

The primary goal of OncoTrack has been the development of computer simulation models, based on an extremely detailed characterization of both tumor and patient. For this, we are using NGS technologies and have so far sequenced the genome/exome of more than 100 tumors (primary tumors and metastases) from an expected total of approximately 300. By sequencing tumors (ideally at different disease progression stages) from the same patient, we expect to gain insight into ways to prevent metastases or at least identify drugs that halt their progression. Comparative analyses of the patient genome and tumor are carried out to identify the specific variants unique to the tumor. Using RNA-Seq, we decipher the transcriptomes of individual tumors in their entire complexity (gene expression, alternative splicing, and allele-specific expression patterns of different classes of transcripts). We also establish the methylation state of the tumors, using a chip-based protocol (Illumina 450k Methylation Arrays).

In order to be able to relate the omics data to the biology of the tumor, we collect information about how the individual tumors respond to therapies. To do this, we attempt to generate 3D cell models and xenografts from all the tumors. In both the in vivo and in vitro systems, models can be successfully established from about 65% of the surgical specimens. The models are then tested against an array of anti-cancer drugs, identifying responders and non-responders, providing the basis for a correlation between omics analyses and the response to the drug. Xenograft and cell transcriptomes and proteomes, and ideally, exomes and methylomes are analyzed before and during treatments (Fig. 1). The 3D cultures allow us to propagate cells in a system that recapitulates important aspects of the plasticity and heterogeneity of the tumor. The resulting cell aggregates or “canceroids” contain substantial numbers of tumor progenitor or so called “cancer stem cells” [68], which we define here as those tumor cells exhibiting known stem cell characteristics, such as the ability to self-renew, expression of stem cell markers, and their multipotency. To validate “cancer stem cells” sub-populations, OncoTrack uses well-established assays such as immunocytochemical detection of known stem cell markers and verification of tumorigenic potential by xenotransplantation.

The mouse xenograft models are generated from primary tumors, metastases, and “cancer stem cells” derived from both primary and metastatic tissue samples. This comprehensive set of models provides a powerful tool for analysis of both novel biomarkers and molecular mechanisms (individualized) of drug response and resistance, as they allow assessment of response to both established and new investigational treatments that cannot yet be tested in clinical trials. The xenograft models not only allow us to measure changes in tumor size and composition but also to follow biochemical or molecular markers of response during the course of a treatment. These models therefore represent a unique resource for the study of tumor response to drug treatment and will be critical as a tool for validation of the predictive power of the ModCell™ model (See section 5.2). In addition, the results obtained will provide insights into the biological processes accompanying adaptation to growth as a xenograft, as well as providing information about the biology of tumor progression during development of metastases.

In addition to tumor tissue, we also collect blood and plasma samples from all patients that we use in experiments designed to assess biomarkers in circulating tumor cells (CTCs) and circulating tumor DNA (ctDNA). The working hypothesis being that CTCs include a critical sub-population of tumors cells that more closely resemble tumor cells in metastases, therefore biomarkers derived from them would be of clinical relevance and widely applicable [69–71]. While both approaches are technically demanding, due to the low numbers of CTCs and low concentrations of tumor DNA present in patients' blood, the potential benefits to the patient of being able to use a blood sample (a so-called “liquid biopsy”) are enormous. Collection of a blood sample, since it is less invasive than a surgical biopsy, can be repeated relatively frequently, allowing longitudinal measurements of biomarkers. The ability to frequently monitor and rapidly detect changes in suitably robust biomarkers will enable the oncologist to adapt treatment to the evolving biology of the individual patient. This should facilitate not only improved early detection of tumors and response to treatment but also early detection of tumor progression and development of metastatic disease.

DNA and RNA are, however, not the only players in the complex biological processes we have to control and model. Proteins and protein modifications play an extremely important role in regulatory processes (as part of post-transcriptional regulation) [72–74]. We are therefore increasingly concentrating our efforts on the analysis of proteins, protein modification states, and protein complexes. At the moment, this relies mostly on a combination of two techniques: Reverse phase protein arrays (RPPAs), a technique in which many tumor or cell extracts are spotted together on chips, in such a way that specific proteins or protein modification states can be detected by extremely specific antibodies [75]. We also use proximity ligation/proximity extension assays [76] that are based on selective formation and detection of DNA fragments when two different antibodies carrying precursors to these oligonucleotides bind in close proximity [77]. The assay permits analysis of up to 92 proteins in parallel, using a homogenous assay requiring only a very small volume (1 μL) of plasma or tissue lysate. In an alternate configuration, detection is achieved by labeling the two antibodies by fluorescent labels, which will only emit light, due to Förster resonance energy transfer (FRET) [78–80], if they are in close proximity. The two techniques can be used to detect low amounts of one or multiple specific proteins (via two appropriately labeled antibodies binding to different sites of the same protein), protein modification states (one antibody binds to the protein and one to the group attached by the modification), or protein complexes (the two antibodies bind to different components of the protein complex), as a biomarker [81–83]. Similar to the detection of specific mutations, or the analysis of the expression of specific RNAs, e.g. by in situ sequencing [83], the protein assays can also be carried out on tissue sections or pathology slides. Fluorescence microscopy can then be employed to collect information not only on the amount of a particular RNA or protein marker in a block of tissue but also the specific amount and location of these components in every cell of the section. Since the anatomical structure of the tumor is preserved in the tissue section, we can readily address the important problem of tumor heterogeneity and also approach questions regarding the microenvironment of the tumor (e.g. the spatial relationship of tumor cells, stroma, cells of the immune system, blood vessels, etc.).

Through this systems-level analysis of molecular and experimental profiling, we have the potential to delineate cause and consequence, correlating molecular profiles with clinical response to facilitate the identification of rarer causal driver mutations and alterations against the background of passenger mutations/alterations, i.e. those that accumulate over time as a consequence of the genetic instability of the tumor, but may have little influence either on the development of the disease or on its response to treatment. At the very least, we gain a fundamental understanding of the genetic variability of neoplasia at the individual case level and a heightened understanding of the biological and molecular properties of tumors and their associated cell populations, providing a platform for investigation of clinically relevant aspects of tumor biology and development of drug resistance.

The parallel application of these varied analyses combines to give us an extremely accurate picture of every tumor and every patient. We can now learn much more about a single tumor than, prior to the human genome project, we knew about the genes and transcripts in a single organism.

### 5.2 What are these computer models?

To be able to develop, manipulate, and execute computer models of biological processes, we have developed PyBioS (pybios.molgen.mpg.de), an object oriented modeling platform [84, 85]. Biological networks representing either the normal biological processes in a specific “normal” cell type, or the modified processes in the tumor cells of a specific patient are assembled from computer “objects” representing the elements (genes, transcripts, proteins and protein modification states, complexes, metabolites, etc.) involved in these biological processes. A biological process represented in the computer by the “normal” components of the “normal” cell types of man should, therefore, also behave “normally” in the computer model, subject to the normal regulatory mechanisms acting in “normal cells”; however, if we introduce the many changes found in the genome, transcriptome, and proteome of the tumor cell into the model, resulting in objects with changed function (e.g. the mutated *RAS* protein), changed abundance (e.g. specific growth factors in autocrine loops), or functions that have become inactivated (e.g. in the case of tumor suppressors), we expect the model to behave like a tumor cell behaves, including the reaction to drugs used in cancer treatment, again represented as objects, which might, for example, interact with specific proteins (protein objects) to form inactive drug–protein complexes.

To compute the consequences of the changes observed in an individual tumor on its behavior, including its response to specific drugs, the PyBioS system translates this object representation automatically into systems of differential equations, which can then be solved numerically. Because many of the parameters (kinetic constants, concentrations of components, which cannot be measured) are unknown, we use a Monte-Carlo approach, drawing unknown parameters from probability distributions reflecting our knowledge (or lack thereof). These parameter vectors are then used to model all conditions we want to compare, e.g. the tumor cell with and without the presence of the different drugs or drug combinations, which could be used to treat the patient.

**Figure 2:**
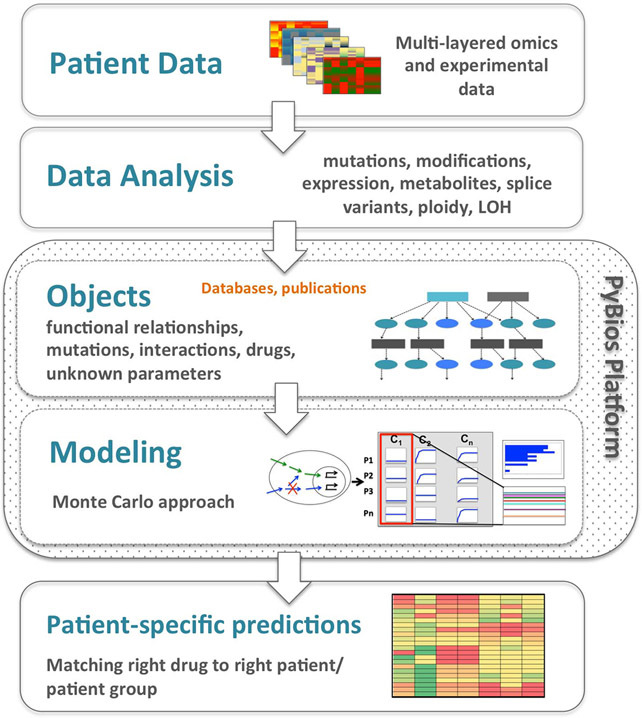
PyBioS and ModCell™. PyBioS (pybios.molgen.mpg.de) and ModCell™ represent an integrative systems biology predictive platform that uses a global network model to predict individual outcomes following virtual treatment. Analysis of patient-derived omics and experimental data from individual tumors is integrated with existing information regarding the consequences of cancer related mutations on a molecular pathway level and their functional effects on the cellular and organism level. Based on a Monte Carlo type strategy, the model samples parameter vectors from a random distribution with statistical significance testing [84, 85]. In this way, information on cellular processes, such as cellular signaling pathways and drug interactions can be integrated, and the model can be applied to investigate the qualitative and quantitative behavior of the underlying biological system given specific perturbations, such as targeted drugs or mutations. The model can predict changes in key components (e.g. expression of specific genes, alteration in abundance of specific growth factors in autocrine loops, and inactivation of tumor suppressors) under different conditions (stimulation with growth factors, mutations, different drugs, and drug combinations at different concentrations), to provide patient-specific predictions and biomarker identification.

The modeling system itself is comprised of two basic components: the modeling infrastructure (PyBioS) and the model itself – ModCell™ – which can be individualized with data from a single patient, and then explored, using the tools provided by PyBioS [85] to create the ModCell™ systems biology integrated platform (Fig. 2).

PyBioS integrates templates for model development and annotation, as a way to automatically take advantage of basic biological knowledge and publically available databases such as Ensembl (http://www.ensembl.org, [86]), Reactome (http://www.reactome.org) and KEGG [87], to automate and speed up model development. The addition of a protein-coding gene to PyBioS will, for example, automatically generate the corresponding transcripts and proteins, based on information from the Ensembl database, and provide default choices for possible reactions they might be involved in. These templates facilitate the identification and integration of new genes, their related proteins, and associated modifications. Gene names can be searched in the PyBioS databases using Ensembl as a reference resource, and the templates keep track of respective mRNA and protein annotation, and automatically create all necessary reactions. This enables the development of well-annotated mathematical/computational models that can be exchanged via Systems Biology Mark Up Language (SBML) [88] and allow the reuse of other published SBML models as provided by BioModels [89].

To further enhance the specificity of model predictions, we also mine public databases, such as Catalogue of Somatic Mutations in Cancer (COSMIC) [90] to identify mutations commonly found in specific cancer types, and catalog these alongside the, as yet, unknown mutations and gene fusions occurring in known cancer genes that have been discovered during NGS analysis of individual OncoTrack tumor samples. The corresponding surrounding signaling networks are also integrated, expanded, or modified as more functional biological data and sequencing data become available.

Data is also retrieved from curated *in house* databases that provide information on cancer mutations and drug interactions. Our current databases hold data on 364 different mutations in 51 oncogenes and tumor suppressor genes, as well as 72 different fusion events in 7 genes, alongside the molecular consequences of these events in the different genes. Our drug catalog holds information relevant to 84 targeted drugs used in cancer treatment (and other diseases) and covers the respective main-target and off-target profiles of the drugs along with known binding parameters of more than 80 different molecular targeted drugs (non/anti-cancer drugs), used for the drug optimization process. In addition, we have implemented inhibitor components that allow us to simulate drug effects, taking more than 95 different drug targets into account.

Overall, the ModCell™ model system provides coverage of more than 620 genes, giving rise to 3397 components (genes, transcripts, proteins, protein modifications, and complexes) that are connected by 5456 reactions.

Through a recent collaboration between Alacris Theranostics (www.alacris.de) and the SAP Innovation Center, Germany (http://www.sap-innovationcenter.com/#), OncoTrack projects can now also take advantage of a much faster differential equation solver, which provides the capacity to run individual modeling scenarios involving multiple conditions (e.g. different drugs or drug combinations and different concentrations); modeling the effect of, e.g. 100 drugs in 10 different doses requires repeated simulation to narrow down statistical uncertainties, generating more than 100 million output values. The improved computing capacity now provides the ability to reduce simulation time of this scenario by a factor of 5000, from 3 h to less than 2 s. By reducing the time from receipt of the samples to a ModCell™-derived treatment recommendation, this throughput speed provides a realistic basis for the eventual clinical application of the system. Moreover, we expect to improve both speed and predictions further, by identifying the regions of the parameter space most predictive for colon tumors, based on a systematic comparison between model predictions and actual results, for example in our xenograft and 3D-cell culture models.

Such a rich and comprehensive catalog of reference data not only provides the means to investigate single cellular pathways but it also offers insights into the cellular cross-talk between pathways, and subsequent knock-on gene regulatory effects. The model provides a goal-orientated framework in which the focus is on individual-by-individual defined mutations/alterations resulting in model components with changed function/abundance and/or gain/loss-of-function through, e.g. complex formation, decay, phosphorylation, dephosphorylation, transcription, translation, and translocation. The precise modeling scenario, i.e. components and interactions, generated are adapted according to the individual tumor, as well as the specific drug that is being modeled.

It must be noted, however, that ModCell™ is still lacking many components that would render it a comprehensive model of a patient; as a comparison, the community-driven implemented Recon2 model (http://humanmetabolism.org/), a large-scale reconstruction of cellular metabolic pathways covers 1789 enzyme-coding genes, 7440 reactions, and 2626 unique metabolites. The curated pathway database currently covers 7327 proteins, 6792 complexes, with 7075 annotated as human. Given that about 25 000 protein-coding genes in the human genome can occur in multiple splice forms, the number of genes integrated in our cellular signaling model is still a fraction (ca. 2.5%) of the overall cellular interaction network; moreover, it is as yet devoid of aspects of the immune system and metabolism, such as cytochrome c, as well as the human microbial milieu. Instead, we focus initially on the “essential” cellular components and reactions, a strategy that has proved to be robust enough to assess the potential of miRNAs as a therapeutic target in colon cancer and helped to identify patient-specific responses to miRNA-based treatments [91].

## 6 Conclusions and future directions

OncoTrack has both long and short-term goals: In the short term, we are developing the detailed molecular characterization of up to 300 tumor samples as the basis of constructing the in silico model that can then be treated (virtually) by drugs or drug combinations of our choice. Implicit in such an approach is the ability to set up virtual clinical trials to identify stratified patient groups that are likely to respond to drug treatment, opening up new avenues for repositioning of approved drugs for specific patient groups or for bringing failed drugs to the market through targeted and much smaller, faster clinical trials.

In the longer term, we expect that deep molecular analyses combined with clinical and pathological information will become routine medical practice, and form the basis of a universal “companion diagnostics” process. Initially, this may be most practical for oncology, but in the long term the approach will also be applicable for many other areas of medicine, prevention, and well being, providing a truly personalized approach to health care.

## Abbreviations

BRAF: v-Raf murine sarcoma viral oncogene homolog B1
EGFR: epidermal growth factor receptor
HER2: human epidermal growth factor receptor 2 (HER2)
KRAS: Kirsten rat sarcoma viral oncogene homolog
NGS: next-generation sequencing
NRAS: neuroblastoma RAS viral oncogene homolog
